# Supplementary material for: Rank-Rankl-Opg Axis in Multiple Sclerosis: The Contribution of Placenta
Source: Cells. 2022 Apr 15;11(8):1357. doi: 10.3390/cells11081357 (PMC9031903; doi:10.3390/cells11081357)
Supplement: Supplementary file 1 [file cells-11-01357-s001.zip › cells-1669732-supplementary.pdf]

RANK-RANKL-OPG axis in Multiple Sclerosis: the contribution of placenta

Sofia Passaponti[1], Leonardo Ermini[1], Giulia Acconci[2], Filiberto Maria Severi[2], Roberta Romagnoli[1], Santina Cutrupi[3], Marinella Clerico[3], Gisella Guerrera[4] and Francesca Ietta[1#]

[1]Department of Life Sciences, University of Siena, Siena Italy;
[2]Department of Molecular and Developmental Medicine, Division of Prenatal Diagnosis and Obstetrics, University of Siena, Siena, Italy;
[3]Department of Clinical and Biological Sciences, University of Turin, Turin, Italy; [4]Neuroimmunology Unit, IRCCS Fondazione Santa Lucia, Rome, Italy.

[#]Corresponding Author: Francesca Ietta; via A. Moro 2, Siena; +39 0577 232370, francesca.ietta@unisi.it

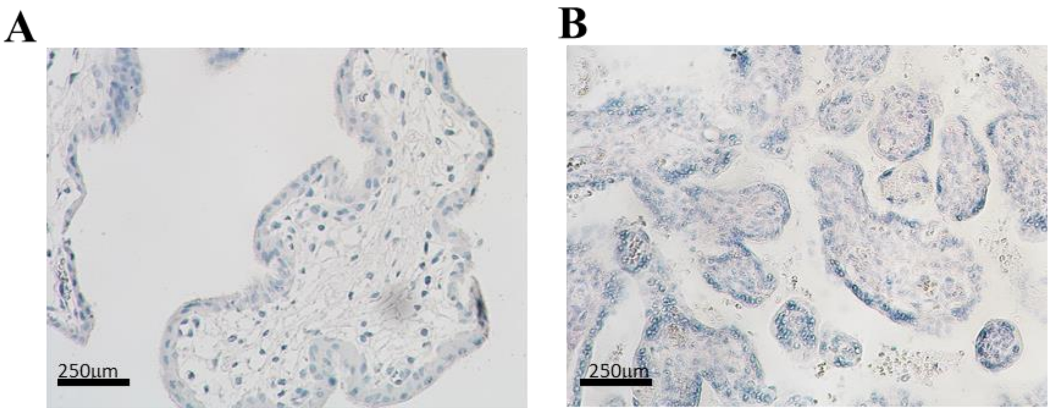

Figure S1. Negative control for OPG immunolocalization. Representative negative controls for OPG immunolocalization in first-trimester (A) and term placenta tissues (B). Immunohistochemistry was performed by substituting the primary antibody by the mouse isotype antibodies.

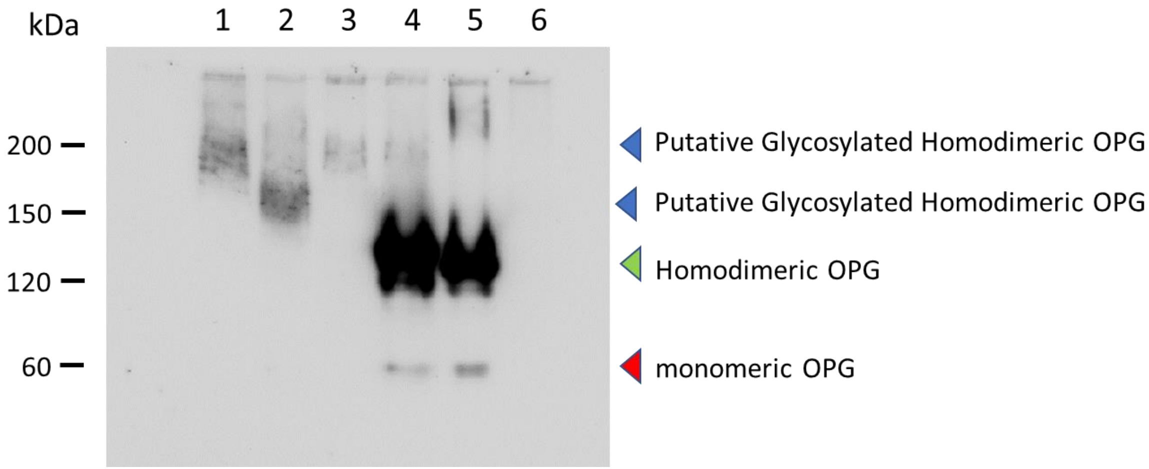

Figure S2. Expression of Homodimeric OPG form. Native western blot was performed in lysates from cell lines known to express OPG (line 1: MCF7; line 2: HT29; line 3: Caco2), in the human

placenta at 38-39 weeks of gestation (line 4: 38 weeks; line 5: 39 weeks) and in cell line not expressing OPG (line 6: A375).

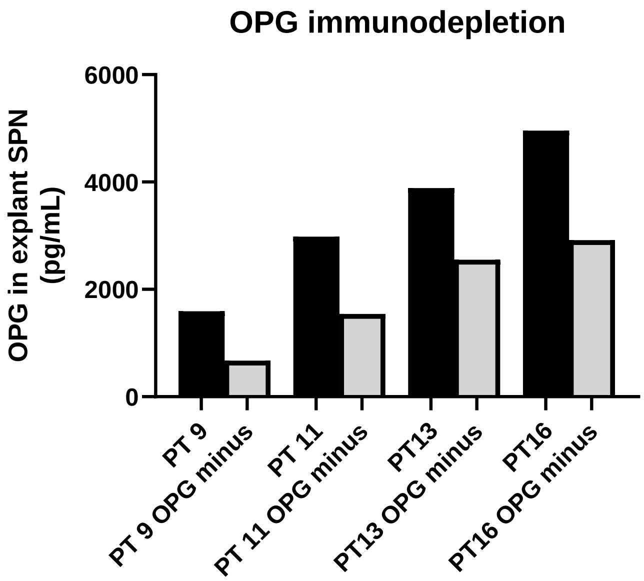

Figure S3. ELISA in SPN before and after OPG immunodepletion. Elisa OPG quantification in n=4 SPN collected after 48 hours of culture. In black the whole SPN and grey is its respective OPG depleted counterpart.
